# Detection of Spliced mRNA from Human Bocavirus 1 in Clinical Samples from Children with Respiratory Tract Infections

**DOI:** 10.3201/eid1904.121775

**Published:** 2013-04

**Authors:** Andreas Christensen, Henrik Døllner, Lars Høsøien Skanke, Sidsel Krokstad, Nina Moe, Svein Arne Nordbø

**Affiliations:** Trondheim University Hospital, Trondheim, Norway (A. Christensen, H. Døllner, L.H. Skanke, S. Krokstad, N. Moe, S.A. Nordbø);; Norwegian University of Science and Technology, Trondheim (A. Christensen, H. Døllner, N. Moe, S.A. Nordbø)

**Keywords:** Human bocavirus, parvovirus, mRNA, splicing, polymerase chain reaction, respiratory tract infections, children, viruses

## Abstract

Human bocavirus 1 (HBoV1) is a parvovirus associated with respiratory tract infections (RTIs) in children, but a causal relation has not yet been confirmed. To develop a qualitative reverse transcription PCR to detect spliced mRNA from HBoV1 and to determine whether HBoV1 mRNA correlated better with RTIs than did HBoV1 DNA, we used samples from HBoV1 DNA–positive children, with and without RTIs, to evaluate the test. A real-time reverse transcription PCR, targeting 2 alternatively spliced mRNAs, was developed. HBoV1 mRNA was detected in nasopharyngeal aspirates from 33 (25%) of 133 children with RTIs but in none of 28 controls (p<0.001). The analytical sensitivity and specificity of the test were good. Our data support the hypothesis that HBoV1 may cause RTIs, and we propose that HBoV1 mRNA could be used with benefit, instead of HBoV1 DNA, as a diagnostic target.

Human bocavirus 1 (HBoV1) is a small nonenveloped virus in the *Parvoviridae* family. It was discovered in human respiratory samples in 2005 (*1*). The virus does not grow in standard cell lines, and diagnosis has mainly been based on DNA detection with PCR. Detection of multiple viruses in HBoV1 DNA–positive airway samples from children with respiratory tract infections (RTIs) has been a characteristic finding in many studies (*2*–*4*). In addition, many healthy children have tested positive for HBoV1 DNA (*2*,*5*); thus, whether the virus actually causes RTIs in children or is just a bystander to other infections has been debated. However, we have shown that the following 3 factors are associated with RTIs: HBoV1 viremia (HBoV1 DNAemia), a high HBoV1 DNA load in nasopharyngeal aspirates (NPAs), and monodection of HBoV1 DNA in NPAs (*5*). In addition, RTIs in HBoV1 DNA–positive children are associated with HBoV1 seroconversion (*6*). This evidence supports a causal relation between HBoV1 and RTIs in children, but DNA-based PCR tests do not seem to diagnose HBoV1 infection accurately. We propose that detection of HBoV1-specific mRNA, as a measure of actively transcribing virus, may be a better method.

The main objectives of this study were to develop a qualitative reverse transcription PCR (RT-PCR) detecting spliced mRNA from HBoV1 and to clarify whether HBoV1 mRNA detection may correlate better than DNA detection with RTIs in children. NPAs and blood samples from a group of children, with and without RTIs, who tested positive for HBoV1 DNA were used for this purpose.

## Materials and Methods

### Samples

HBoV1 DNA–positive NPA samples from an ongoing project on RTIs in children 0–16 years of age were used for evaluation of the test (*5*). In particular, 161 NPA samples collected at admittance from 161 children at the Department of Pediatrics, St. Olav’s Hospital, Trondheim University Hospital (Trondheim, Norway), during June 2007–June 2010 were included. A blood sample was also available for 63 of the children. All samples had been stored at −70°C.

### Children with RTIs

Of the 161 HBoV1 DNA–positive NPA samples, 133 were from children with RTIs. Median age was 17 months (range 3 months–5 years) and 60% were boys. They were classified as having either lower (86 children) or upper (47 children) RTI (LRTI; URTI). LRTI was diagnosed in the presence of dyspnea, signs of lower airway obstruction (wheezing, retractions), and/or a chest roentgenogram with positive results (infiltrates, atelectasis, air trapping). URTI was diagnosed when rhinitis, pharyngitis, and/or otitis media was found without signs of LRTI. In addition, 3 children with RTIs, admitted during winter 2011–12, were followed up on 3 occasions, each over 2 months.

### Children without RTIs (Controls)

Twenty-eight HBoV1 DNA–positive NPA samples were collected from a group of children who were admitted for elective surgery and who had exhibited no signs or symptoms of RTI during the previous 2 weeks. The children were included prospectively during the same period in 2007–2010. Median age was 31 months (range 15 months–6 years), and 70% were boys.

### Tests for Other Respiratory Agents

All NPA samples from patients and controls were also tested with PCRs for adenovirus, coronavirus (OC43, 229E, and NL63), enterovirus, parechovirus, human metapneumovirus (HMPV), influenza A and B viruses, parainfluenza virus types 1–4, respiratory syncytial virus (RSV), rhinovirus, *Bordetella pertussis*, *Chlamydophila pneumoniae*, and *Mycoplasma pneumoniae*. The PCRs were in-house, real-time assays with TaqMan probes (Roche Diagnostics, Basel, Switzerland) (*5*). The analyses were conducted as part of the daily laboratory routine and performed within 24 hours after sample collection. The target for the HBoV1 DNA PCR was the nuclear phosphoprotein-1 gene. This PCR has been described (*4*). A semiquantitative approach was chosen, and a cutoff value of 10^6^ copies/mL was used to distinguish between high and low HBoV1 DNA load in NPAs.

### Spliced HBoV1 mRNA-PCR

**Figure 1:**
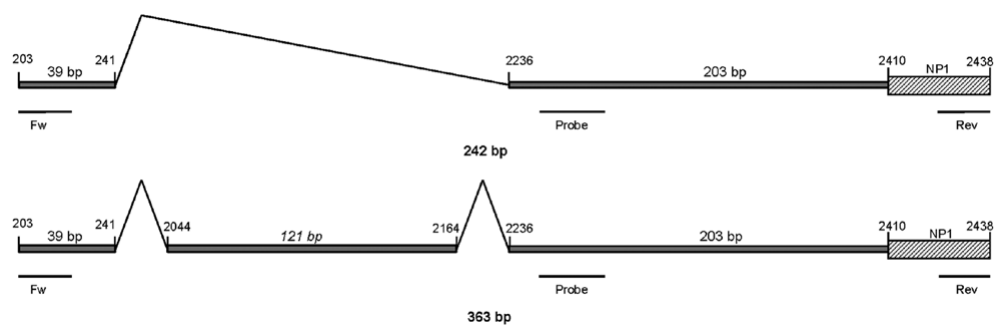
Schematic representation of the 2 human bocavirus 1 (HBoV1) mRNA PCR products, illustrating alternative splicing. Positions of primers and probe are shown. The total length of the upper product is 242 bp, and the length of the lower is 363 bp (reference sequence: GenBank accession no. NC007455).

We developed a real-time RT-PCR on the basis of TaqMan technology (Roche). The following primers were designed: forward 5′-CGGCGAGTGAACATCTCTGGA-3′ (positions 203–223) and reverse 5′-TGCTTGTCTTTCATATTCCCT-3′ (positions 2438–2418). The estimated PCR product spanned a spliced segment from positions 241 to 2236 of the complete genome for HBoV1 (GenBank accession no. NC007455), which gives a theoretical PCR product of 242 bp and an alternative product, including a short segment from positions 2044 to 2164, yielding a product of 363 bp (Figure 1). These estimations were based on in vitro studies performed by Chen et al. (*7*). The probe targeting the untranslated region upstream of the nuclear phosphoprotein-1 gene had the following sequence: 5′-FAM-TGTCCACCCAAGAAACGTCGTCTAA-TAMRA-3′ (positions 2295–2319). The PCR for every sample was also run without reverse transcription to test for potential unspecific reactions with viral DNA. The theoretical PCR product from HBoV1 DNA would be 2,236 bp in length, which is too long for amplification by real-time PCR under normal conditions.

Total DNA and RNA were extracted by using NucliSens easyMag extractor (bioMérieux, Marcy l’Etoile, France), and reverse transcription was carried out with Universal RiboClone random primers (Promega, Fitchburg, WI, USA) and M-MLV Reverse Transcriptase (Life Technologies Corp., Carlsbad, CA, USA) at 37°C for 60 min. followed by 94°C for 10 min. The PCR was performed for 45 cycles at 95°C for 5 s., 55°C for 10 s, and 72°C for 20 s.

Amplification efficiency was calculated by using the formula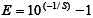, where *S* is the slope of the standard curve. A human DNA PCR (specific for the γ-glutamyltransferase light chain 1 gene on chromosome 20) was used as amplification control (*8*). Nucleic acid extract from a clinical sample positive for RSV was used as cDNA control. To make sure that mRNA had not been degraded during storage, we used an RT-PCR to detect human β actin mRNA (*9*).

RNA stability was studied by using clinical NPA samples and nucleic acid extracts from the easyMag extractor (bioMérieux). Four clinical NPA samples collected within the previous 2 hours were stored for 0, 1, 3, and 5 days at 4°C before nucleic acid extraction and testing with the HBoV1 mRNA PCR. One NPA sample was stored at room temperature and tested likewise. Two other clinical NPA samples were frozen and thawed 0, 1, 2, and 4 times before extraction and testing with the HBoV1 mRNA PCR (3 times was skipped to save NPA material). Furthermore, HBoV1 mRNA PCR results from 3 HBoV1-positive NPA samples stored at −70°C for 3 years were compared with nucleic acid extracts from the same samples stored under equal conditions for the same period. This was done to determine whether the stability of RNA in clinical NPA samples added to virus transport medium was comparable to the stability of RNA in nucleic acid extracts from the easyMag at this temperature. Relative changes in RNA load were measured by comparing logarithmically transformed cycle threshold values (Ct values) obtained from the same experiment.

Quantitative standards for the real-time HBoV1 mRNA PCR were made by cloning a plasmid (pCR4-TOPO; Life Technologies Corp.) containing the PCR product. The amount of nucleic acid was measured, and serial dilutions covering a range of 7 logs were made to measure the analytical sensitivity of the HBoV1 mRNA PCR.

Analytical specificity was evaluated by using cDNA from NPA samples positive for all respiratory agents included in the study. cDNA from NPA samples containing viruses that can be reactivated in the respiratory tract were also included (i.e., herpes simplex virus, cytomegalovirus, Epstein-Barr virus, and human herpes virus 6). Finally, cDNA from NPA samples positive for the more closely related parvovirus B19 and cDNA from fecal samples positive for human bocaviruses 2 and 3 (HBoV2 and HBoV3) were tested. The primers and probe described by Kantola et al. were used for detection of HBoV2 and HBoV3 (*10*). Two samples positive for each agent were used, and all samples had undergone extraction within 2–20 hours after sample collection. Sequence analysis on the PCR products was performed by using the BigDye Terminator Cycle sequencing method and the ABI Prism 3130 Genetic Analyzer (Applied Biosystems, Foster City, CA, USA).

### Statistical Analysis

Statistical analysis was by χ^2^ test for categorical variables and Student *t* test for continuous variables. Multiple logistic regression analysis was used to evaluate the association between detection of HBoV1 mRNA and LRTI, controlling for differences in age, sex, and the presence of other viruses among case-patients and controls. We report the odds ratio (OR) with 95% CIs and the corresponding p value as a measure of the strength of the association. All analyses were performed by using IBM SPSS Statistics version 19.0 (SPSS Inc., Chicago, IL, USA). 

## Results

### Spliced HBoV1 mRNA PCR

**Figure 2:**
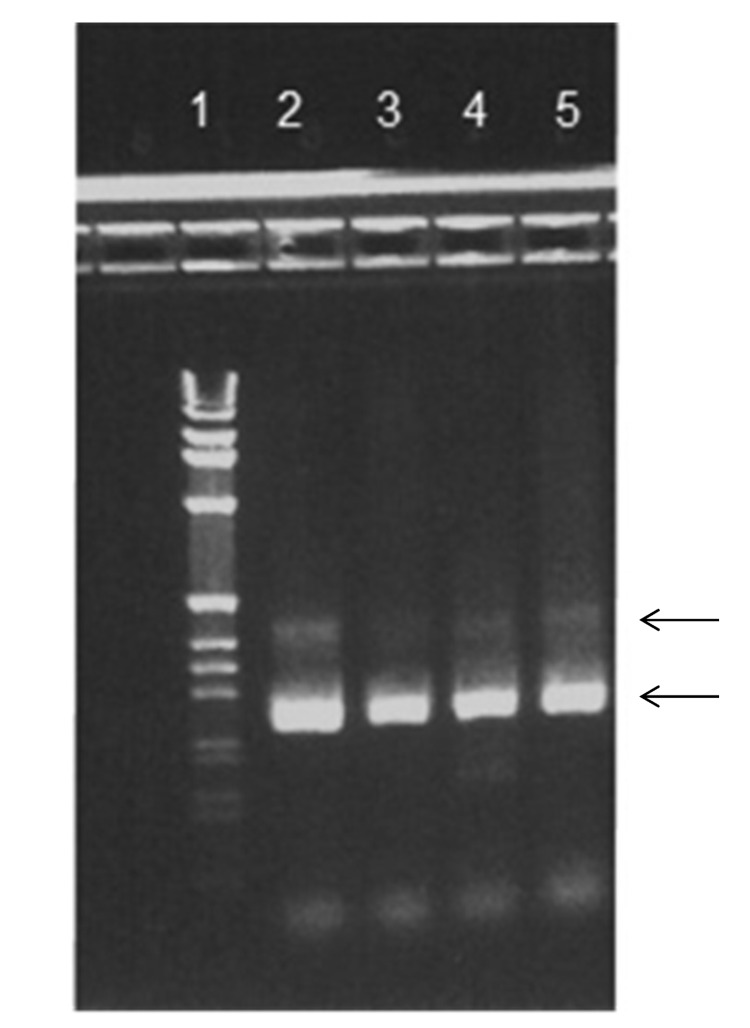
Agarose gel stained with ethidium bromide. Reverse transcription PCR products from 4 patients are shown in lanes 2–5 and 1-kb DNA Ladder (Life Technologies Corp., Carlsbad, CA, USA) in lane 1. Arrows indicate 2 bands corresponding to ≈250 and ≈400 bp.

HBoV1 mRNA was detected in 33 of the 161 HBoV1 DNA–positive NPA samples (Table 1). Gel electrophoresis showed that primarily 2 PCR products were amplified with sizes of ≈250 bp and ≈400 bp (Figure 2). Sequence analysis showed that they were spliced products with either 1 or 2 introns cut out as expected (schematically illustrated in Figure 1). The exact product sizes were 242 bp and 363 bp. Direct PCR analysis for HBoV1 mRNA on the nucleic acid extracts, without initial reverse transcription, was negative for 30 of 33 NPA samples. For the remaining 3 samples, however, weak signals were detected. These 3 samples had very high HBoV1 DNA loads (range 4 × 10^8^ copies/mL to >10^10^ copies/mL), and were also strongly positive by the HBoV1 mRNA PCR after cDNA synthesis. The products of the 3 PCRs that were done without cDNA synthesis were sequenced. Product sizes were 145, 261, and 457 bp, and sequence analysis showed gaps at different positions, all of them lacking splice site characteristics (data not shown).

**Table 1:** HBoV1 mRNA PCR results in NPAs from children with and without RTIs, Norway, 2007–2010*

Sample source	Total no.	No. (%) HBoV1 mRNA^+^	No. (%) HBoV1 mRNA^–^	p value
Children with RTIs	133	33 (25)	100 (75)	p<0.001
Controls (without RTIs)	28	0	28 (100)	
Children with LRTIs	86	27 (31)	59 (69)	p = 0.02
Children with URTIs	47	6 (13)	41 (87)	

*HBoV1, human bocavirus 1; NPAs, nasopharyngeal aspirates; RTIs, respiratory tract infections; LRTIs, lower RTIs; URTIs, upper RTIs.

Amplification efficiency of the HBoV1 mRNA PCR was calculated on the basis of dilutions of both the nucleic acid extract and cDNA. It was measured to 100% in both cases, indicating a high efficiency of both the PCR and cDNA synthesis (data not shown). The assays’ reportable range was from 500 copies/mL (10 copies/reaction) to 10^10^ copies/mL.

Results of the β-actin PCR performed after DNase treatment were positive for all samples studied. Results of the HBoV1 mRNA PCR were negative for all other respiratory agents, herpesviruses, and parvoviruses tested.

### mRNA Stability

The HBoV1 mRNA load in NPA remained stable for 5 days at 4°C. At room temperature, it was unaltered after 24 h but was reduced by 1 log after 3 days and by 1.5 log after 5 days. Freezing and thawing of the NPA samples once or twice did not affect yield, but after 4×, it was reduced by ≈0.5 log. For 3 NPA samples that had been stored at −70°C for 3 years, the results were equal for both the nucleic acid extract and the original sample. HBoV1 mRNA PCR results for nucleic acid extracts were stable for weeks when samples were stored at 4°C (samples stored for up to 8 weeks were tested; data not shown).

### Performance of Spliced HBoV1 mRNA PCR on Samples 

First, we compared the rates of positive test results for HBoV1 mRNA among children with positive results for HBoV1 DNA, with and without RTIs. Only one fourth of the patients and none of the controls had test results positive for HBoV1 mRNA (Table 1). More children with LRTI (27/86 [31%]) than with URTI (6/47 [13%]) had positive test results for HBoV1 mRNA (Table 1). After we adjusted for age, sex, and presence of other viruses, this difference persisted (OR 3.5, 95% CI 1.3–9.8, p = 0.02).

**Figure 3:**
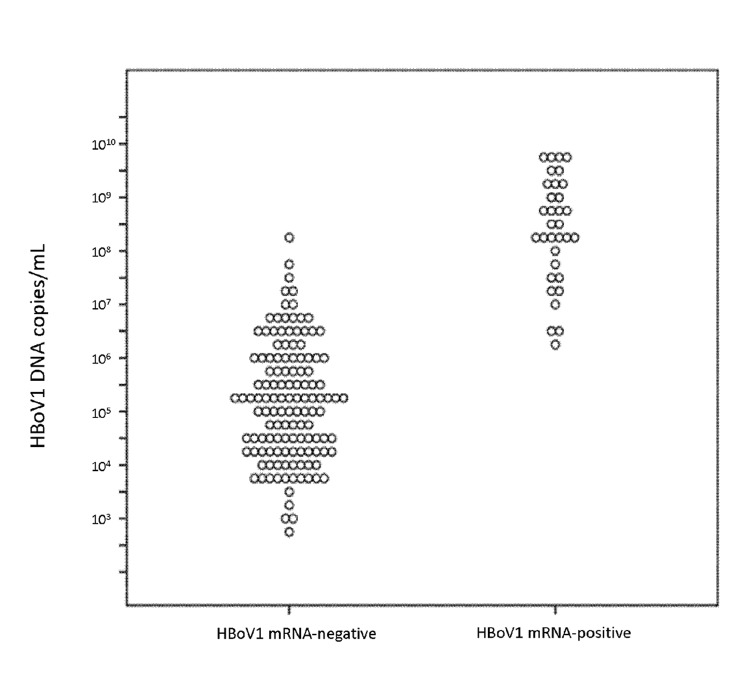
Distribution of human bocavirus 1 (HBoV1) DNA loads in nasopharyngeal aspirates either positive (n = 33) or negative (n = 128) for HBoV1 mRNA. Each dot indicates 1 sample.

We previously found that 3 factors (HBoV1 DNAemia, high HBoV1 DNA in NPAs, and monodection of HBoV1 DNA in NPAs) were each associated with RTIs in children (*5*). In the present study, these factors were strongly associated with a positive test result for HBoV1 mRNA (Table 2). The close relationship between HBoV1 DNA load and HBoV1 mRNA detection in NPAs is also illustrated in Figure 3. Of the 100 RTI patients who were negative for HBoV1 mRNA, 75 were positive for >1 other respiratory viruses. Twenty-eight (37%) of these children were infected with the highly pathogenic RSV. Distribution of the viruses most commonly co-detected with HBoV1 is shown in Table 3.

**Table 2:** HBoV1 mRNA PCR results in NPAs in relation to HBoV1 DNAemia, a high HBoV1 DNA load, and monodection of HBoV1 DNA, Norway, 2007–2010*

Factor	Total no.	No. (%) HBoV1 mRNA^+^	No. (%) HBoV1 mRNA^–^	p value
HBoV1 DNAemia, n = 63	17	13 (77)	4 (23)	p<0.001
No HBoV1 DNAemia	46	5 (11)	41 (89)	
HBoV1 DNA load, n = 161				
>10^6^ copies/mL	59	33 (56)	26 (44)	p<0.001
<10^6^ copies/mL	102	0	102 (100)	
>10^8^ copies/mL	18	17 (94)	1 (6)	p<0.001
<10^8^ copies/mL	143	16 (11)	127 (89)	
Monodection of HBoV1 DNA, n = 161	43	14 (33)	29 (67)	p = 0.022
Multiple virus detections	118	19 (16)	99 (84)	

*HBoV1, human bocavirus 1; NPAs, nasopharyngeal aspirates; HBoV1 DNAemia, HBoV1 viremia.

**Table 3:** Most commonly co-detected viruses in NPAs from children with HBoV1 DNA, distributed by presence of RTI and HBoV1 mRNA, Norway, 2007–2010*

**Virus**	**HBoV1 DNA+ children with RTIs**			**HBoV1 DNA+ controls**	
	No. (%) HBoV mRNA+, n = 33	No. (%) HBoV1 mRNA–, n = 100†		HBoV mRNA+, n = 0	HBoV1 mRNA–, n = 28†
**Respiratory syncytial virus**	4 (12)	28 (28)		–	0
**Rhinovirus**	7 (21)	25 (25)		–	10 (36)
**Enterovirus**	5 (15)	24 (24)		–	15 (54)
**Adenovirus**	1 (3)	20 (20)		–	9 (32)


*NPAs, nasopharyngeal aspirates; HBoV1, human bocavirus 1; RTIs, respiratory tract infections; +, positive; –, negative. **†Triple and quadruple infections were common, and percentages within the columns may therefore add up to >100%.**

### Follow up of 3 Children with RTIs during Winter 2011–12

During winter 2011–12, sequentially collected samples from 3 HBoV1 DNA–positive children made it possible to gain some information about changes in HBoV1 mRNA and HBoV1 DNA over time. One of these children (boy 1) was a 2-year-old boy with cerebral palsy who had been admitted with bronchiolitis. On admission, he had HBoV1 DNAemia and analysis of NPAs showed that he had 1) a high HBoV1 DNA load, 2) monodection of HBoV1 DNA, and 3) positive HBoV1 mRNA PCR results. He recovered slowly, and after 10 days a new NPA sample was taken. The HBoV1 DNA load was still high, but the results for the HBoV1 mRNA PCR were negative. Two months later, his NPAs still were positive for HBoV1 DNA and negative for HBoV1 mRNA. The other patients were two 1.5-year-old boys (boys 2 and 3), who had been included in an HMPV follow-up study. Their NPA samples became positive for HBoV1 DNA 1 week after HMPV infection was diagnosed. The initial samples were negative for HBoV1 DNA. The clinical condition was unaltered for both when HBoV1 DNA appeared. HBoV1 DNA loads reached moderate levels for boy 2 and high levels for boy 3, but for both boys, the PCRs were negative for HBoV1 mRNA in 2 consecutive NPA samples taken 5 days apart. Unfortunately, no blood samples were taken from these 2 boys.

## Discussion

We report here the development of a robust PCR for detection of spliced mRNA from HBoV1. We found that that HBoV1 mRNA correlated significantly better with RTIs in children than did HBoV1 DNA, indicating that this PCR may diagnose HBoV1 infection more accurately than PCR for HBoV1 DNA.

Splicing is a process specific for mRNA synthesis, and with use of a primer pair spanning an intron, spliced viral mRNA should be specifically detected within the frame of the familiar and robust RT-PCR. This diagnostic technique has been used to diagnose parvovirus in dogs and may also be an option for diagnosing HBoV1 infections in humans (*11*). Furthermore, detection of mRNA is routinely used for diagnosing human papillomavirus infections and has been studied for diagnosing human herpesvirus 6 and HIV infections (*12*–*15*). However, the mRNA tests for these viruses have been based on either specific mRNA extraction, nucleic acid sequence–based amplification technology, or pretreatment with DNase. The advantage with our approach is that no pretreatment, other than cDNA synthesis, is needed. The procedure is performed as a regular RT-PCR with standard equipment and will be easy to use in most routine laboratories. The analytical performance of the test was good with high analytical specificity and sensitivity.

The probe target was chosen to detect the 2 products (Figure 1) and thereby to maximize analytical sensitivity. A probe spanning the spliced segment between positions 241 and 2236 would have been an alternative approach, ensuring specific detection of mRNA spliced at this exact location (Figure 1). However, because our results indicated high specificity with the chosen probe, we did not develop this approach further.

Previously, RNA molecules were believed to have short half-lives because RNases may be present everywhere and easily degrade RNA. Recent studies, however, have suggested that mRNA may be stable when molecules are kept in the original biologic material (*16*,*17*). We found that the mRNA content in NPA samples was stable during a 5-day period at 4°C. This stability suggests that NPA samples kept in a refrigerator and processed within 1–2 days, which is standard in our laboratory, are safe to use for HBoV1 mRNA testing.

In 3 samples that had strong signals in the HBoV1 mRNA test, a weak signal was detected also without prior cDNA synthesis, evoking the question of whether HBoV1 DNA could give false-positive reactions in the HBoV1 mRNA test. The PCR was designed so that the theoretical DNA product would be 2,236 bp—too large for amplification to occur in a regular, real-time PCR. For this reason, the PCR products were expected to result from recombination events. Gel and sequence analysis showed that all 3 products had different sizes, ranging from 145 to 457 bp. Moreover, no common sequence profiles were found near the gap junctions, which seemed to be located at random. Homologous recombination, a concentration-dependent process, may explain this phenomenon because it occurred only in NPA samples with extremely high levels of HBoV1 DNA. We speculate that the PCR products might have been subpopulations of nonviable HBoV1 mutants that appeared when virus replication was at its highest. However, the specificity of the test was not affected because it happened only in patients with very high viral DNA loads and strong HBoV1 mRNA signals.

In addition to being a diagnostic test, this method may be used to gain information on HBoV1 transcription in vivo. Our data confirmed previous in vitro results on the splicing pattern at the 5′ end of the HBoV1 genome (Figure 1) (*7*,*18*).

For evaluation of the HBoV1 mRNA test, we were able to use available clinical samples from children with or without RTIs who had been tested for 18 respiratory agents and had HBoV1 DNA in NPAs (*5*). None of the children without RTIs had detectable HBoV1 mRNA. Because mRNA is a marker of active viral transcription, this finding indicates that the 28 asymptomatic children carried inactive HBoV1 or HBoV1 with low activity. The strong association found between active HBoV1 transcription and RTI in children supports the hypothesis that HBoV1 may cause RTIs. The hypothesis is further supported by the associations found between HBoV1 mRNA and the 3 factors: HBoV1 DNAemia, high HBoV1 DNA load in NPAs, and monodection of HBoV1 DNA—all factors strongly related to RTIs in children (*5*,*6*,*19*). In addition, the fact that HBoV1 mRNA was more frequently detected in children with LRTIs than with URTIs indicates that LRTI is a prominent manifestation of HBoV1 infection.

Only one fourth of the HBoV1 DNA–positive children with RTIs had detectable HBoV1 mRNA. Similar findings were recently reported by Proenca-Modena et al. (*20*). The absence of HBoV1 mRNA in most of the children with RTI may indicate that these children did not have a clinical HBoV1-infection, despite positive test results for HBoV1 DNA. Indeed, other respiratory viruses were frequently detected among the children who had a negative HBoV1 mRNA test result; RSV accounted for one third of infections.

The previously mentioned strong relation between HBoV1 DNA load in NPAs and HBoV1 mRNA is illustrated in Figure 3. It shows 2 distinct populations with little overlap, and good discrimination between HBoV1 mRNA–positive and –negative samples can be achieved with cut-off values from 10^6^ to 10^7^ HBoV1 DNA copies/mL. We suggest that, for clinical purposes, HBoV1 mRNA is more accurate than HBoV1 DNA in diagnosing active HBoV1 infection, but a high HBoV1 DNA load (>10^7^ copies/mL) may also be useful in diagnosis.

Previously, HBoV1 has been found to persist in NPAs for many months (*21*–*23*). The molecular basis for this persistence is largely unknown, but 2 recent studies have given evidence in support of persistent circular HBoV episomes (*24*–*26*). The NPA samples in our study which were negative for HBoV1 mRNA and positive for HBoV1 DNA could be from patients with past HBoV1 infections who were still shedding viral DNA. Boy 1, who was followed up during winter 2011–12, may illustrate this. Results of PCR on NPA samples from this boy were positive for HBoV mRNA only for a short period (<10 days), coinciding with the acute symptomatic infection, whereas HBoV1 DNA persisted for months. An alternative hypothesis might be that the samples negative for HBoV1 mRNA and positive for HBoV1 DNA were from children with a latent HBoV1 infection. The findings in boys 2 and 3, who were followed up during the same winter, may support this hypothesis. HBoV1 DNA in NPAs appeared during an ongoing HMPV infection in both children, but results of PCR for HBoV1 mRNA remained negative in 2 consecutive samples. The lack of detectable HBoV1 mRNA may indicate that HBoV1 did not play a role in these infections. Release of latent HBoV1 DNA from cells disrupted by inflammation caused by HMPV may be a better explanation. More longitudinal studies, including serologic analyses, are needed to further study these relationships.
